# What is being done to respond to the rise of chronic diseases and multi-morbidity in Czechia, Hungary, Poland, and Slovakia?

**DOI:** 10.3389/fpubh.2022.1082164

**Published:** 2023-01-16

**Authors:** Anna Sagan, Iwona Kowalska-Bobko, Lucie Bryndová, Martin Smatana, Ihor Chaklosh, Pétér Gaál

**Affiliations:** ^1^European Observatory on Health Systems and Policies, London, United Kingdom; ^2^London School of Economics and Political Science, London, United Kingdom; ^3^London School of Hygiene and Tropical Medicine, London, United Kingdom; ^4^Institute of Public Health, Jagiellonian University, Krakow, Poland; ^5^Faculty of Social Sciences, Charles University, Prague, Czechia; ^6^Faculty of Public Health, Slovak Medical University, Bratislava, Slovakia; ^7^Health Services Management Training Centre, Semmelweis University, Budapest, Hungary

**Keywords:** chronic disease, multi-morbidity, care coordination, integrated care, Czechia, Hungary, Poland, Slovakia

## Abstract

Although countries in central and eastern Europe (CEE) have relatively younger populations compared to the West, their populations are often affected by higher prevalence of chronic conditions and multi-morbidity and this burden will likely increase as their populations age. Relatively little is known about how these countries cater to the needs of complex patients. This Perspective piece identifies key initiatives to improve coordination of care in Czechia, Hungary, Poland, and Slovakia, including some pioneering and far-reaching approaches. Unfortunately, some of them have failed to be implemented, but a recent strategic commitment to care coordination in some of these countries and the dedication to rebuilding stronger health systems after the COVID-19 pandemic offer an opportunity to take stock of these past and ongoing experiences and push for more progress in this area.

## Introduction

More than one out of three adults in the European Union (EU) report having a long-standing (chronic) illness or health problem, and an increasing proportion of the chronically ill people suffer from multi-morbidity, having two or more chronic conditions (1, 2). Multi-morbidity is most common among older people, with reported prevalence of up to 65% in people aged 65+ and up to 85% in people aged 85+ (2, 3). Increasing life expectancy means that the number of people afflicted with multiple health problems is likely to increase too.

Due to a variety of socio-economic, technological, demographic, and epidemiological factors, these problems have been more pronounced in Western Europe, leading to the emergence of various strategies and approaches to improve care for people with chronic conditions (2). So far these have been focused on specific diseases and medical specialties, including type 2 diabetes, asthma/chronic obstructive pulmonary disease (COPD), cardiovascular diseases, cancer, and mental health problems, and hardly any have targeted multi-morbidity (2, 4). However, a variety of approaches has emerged to improve the organization and coordination of care for patients with complex chronic health needs, often involving primary care practices and focusing on multi-professional cooperation to better manage individual cases (2).

Countries in central and eastern Europe (CEE) have relatively younger populations compared to the West, but their populations often report higher prevalence rates of chronic conditions, such as diabetes, asthma, COPD, hypertension, and depressive disorders, and of multiple conditions (2), and population aging means this burden may further increase. Relatively little is known about how (and even if) these countries are responding to the changing disease patterns and the increasing burden of chronic diseases and multi-morbidity. In this context, this Perspective piece seeks to review the key efforts undertaken in Czechia, Hungary, Poland, and Slovakia in response to the rising prevalence of chronic conditions in their populations.

## The hasty return to social health insurance after 1989

Health systems of Czechia, Hungary, Poland, and Slovakia followed similar historical trajectories. All four established Bismarckian-style social health insurance systems in the second half of the 19^th^ century (5–8). After the Second World War, all four adopted the Soviet-style centralist system of state health care financed from general taxation, only to return to the Bismarckian model as quickly as possible after the fall of Communism. However, the 45 years spent under the Semashko system have left a legacy that is still visible to this day, including the relatively large numbers of hospital beds and the relative weakness of primary health care (PHC).

Under the Soviet model, health care was usually delivered by physicians with narrow specializations and provision was dominated by hospitals (9). Public polyclinics were the cornerstone of health care provision in the community, uniting primary and outpatient specialist services in one location and serving specified geographical (mostly urban) areas (10). The advantage of this set up was immediate access to specialists for the patients and opportunities for closer cooperation between primary and secondary care physicians. However, co-location of services was not accompanied by corresponding coordination mechanisms and this, together with the outdated facilities and equipment and low salaries, meant that care provided in polyclinics was of poor quality (10). Despite some initial health gains, mainly driven by the eradication of epidemic diseases thanks to the laboratory-based SANEPID (sanitary-epidemiological) service, the system proved unfit to cope with new challenges, including the rise of lifestyle-related non-communicable diseases (11).

The hasty return to social health insurance after 1989 was largely motivated by politics and ideology (9, 11, 12). It was accompanied by wide-ranging reform efforts, which included decentralization of health care administration, reducing the size of the hospital sector, expansion of private provision, especially in PHC, development of family medicine and general practice, changes in provider payment (with introduction of capitation payment in PHC and diagnosis-related groups (DRGs) in hospital care, counting among the key changes), strengthening of public health and improving quality of care (9). The speedy departure from the Soviet model meant that what came next was not always well thought through and some aspects of the old system, which perhaps could have been capitalized on and improved, were outright abandoned. For example, the dissolution of the centrally managed polyclinics resulted in many independent solo practices and ran counter to the trend to establish group practice and improve coordination between primary, specialist, long-term care and public health services that was emerging in much of western Europe (9). To this day, primary care remains relatively weak in many CEE countries, with narrow roles (e.g., limited use of minor surgery and diagnostics) and less prestige compared to specialist care, which, combined with limited gatekeeping, means that it is often bypassed in practice [see e.g., (5–8, 13)]. At the same time, the introduction of capitation fee as the main mode of payment for PHC has been blamed for under-provision of primary care services, and a rise in referrals to specialist care, while the introduction of DRGs in hospital care was criticized for obstructing coordination (9). At the start of 1990s, the health systems in CEE were largely based on an acute, episodic model of care concentrated in hospitals and were ill-equipped to deal with chronic diseases and multi-morbidity (9), and the reforms of the early the 1990s did little to rectify this situation. This does not mean, however, that the problem was not recognized, and all four countries have made attempts to optimize care pathways for patients with multiple chronic conditions.

## A pioneering but failed care coordination initiative in Hungary

Hungary's Care Coordination System (CCS), introduced in 1998, was a truly pioneering initiative in the area of care coordination, not only in Hungary but also at the European level. The idea behind the CCS was to provide financial incentives to health care providers to coordinate their activities across levels of care for a population living in a geographically defined area (initially up to 200,000 people) (4, 14, 15). Hospitals, independent polyclinics, or groups of family doctors could become care coordinators and manage a virtual budget, based on weighted capitation, set by the National Health Insurance Fund Administration (NHIFA). If, at the end of the year, the total cost of provided care was lower than this virtual budget, the coordinator would receive the difference and could use it for investments or other purposes (e.g., to increase salaries). To achieve maximum efficiency improvements and cost savings, all types of care coordinators had to collaborate with other health care providers in their region (social care providers were not part of the initiative but involving them was not prohibited either) to optimize patient pathways, for example by ensuring provision of appropriate outpatient care to reduce avoidable hospital admissions. Patients retained the right to choose providers outside of the CCS, but all payments made to these providers would be deducted from the virtual budgets. This limited incentives for the CCS to achieve savings by undertreating patients and meant that coordinators had financial responsibility for care received by all patients in their area, including care received outside of the CCS.

Various case management models were developed within the CCS to achieve savings. Both hospitals and independent polyclinics reported their activity data to the NHIFA for reimbursement purposes and care coordinators, using patients' social insurance identification numbers, could retrospectively reconstruct and optimize care pathways at the level of individual patients. At the same time, existing disease management programmes (DMPs) were also embedded into the CCS. These were originally developed with the support from the pharmaceutical or medical devices industries and were provided either within specialist outpatient units (e.g., diabetes care) or in dispensaries (e.g., in pulmonary dispensaries for asthma care). Coordinators would identify high risk individuals and include them in DMPs. Self-management was also encouraged within the CCS, with patient education and 24/7 consultation services provided by highly qualified nurses, including within the specific DMPs. As such, the CCS model was very comprehensive, catering to population groups of varying degrees of need, from supporting disease prevention in the general population to supporting individuals with highly complex needs, akin to the Kaiser Permanente approach in the USA and other population-based initiatives (16, 17) (Box 1).

Box 1 **Coordinated care initiatives in Czechia, Hungary, Poland, and Slovakia according to the risk profiles of the populations they serve.**

Kaiser Permanente is a US non-profit health maintenance organization with a long track record in improving integration of health services (16). This is supported by tailoring provision to the needs and risk profiles of different groups of patients, as depicted in Figure below (the so-called “Kaiser Triangle” or “Kaiser Pyramid”). There is thus a strong emphasis on disease prevention for the entire population (bottom layer of the triangle) and self-management (second layer); disease management and care pathways are available for patients with common conditions (third layer), and case management is offered to patients with the most complex needs (top layer).

Comparison of care coordination initiatives is not easy, including because similar programmes can use different nomenclatures in different countries. The Kaiser Triangle described above offers a simple framework for comparing these initiatives, by focusing on the different populations they serve that correspond to the different layers of the triangle. Some of the key care coordination initiatives pursued in Czechia, Hungary, Poland, and Slovakia, are shown in the figure below.

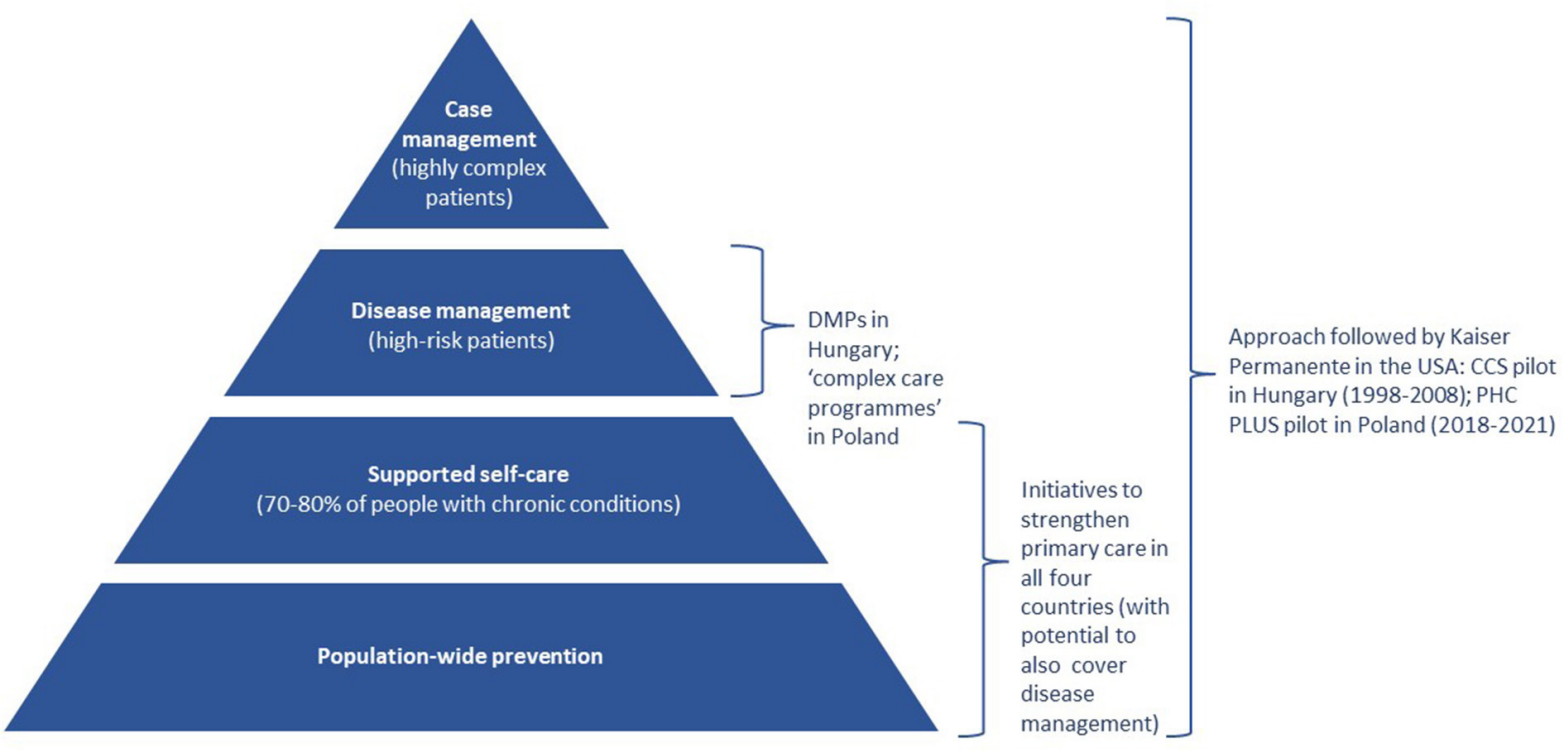

Key coordinated care initiatives in Czechia, Hungary, Poland, and Slovakia mapped onto the Kaiser Triangle. Source: Authors based on (17).

The model was gradually rolled out to cover over 2.2 million inhabitants (over 20% of the population) by 2005. However, reform priorities soon changed and the new government (2006–2010) focused on introducing managed competition in the public health insurance system by replacing the NHIFA with multiple, partially private health insurers that were expected to compete for enrollees on the quality of services (6). This did not preclude the continuation of the CCS model, *per se*, but the government preferred to give the responsibility for care coordination to the private health insurance companies who could then implement coordination tools of their own choice. Political attention shifted to this new model, and despite the fact that the introduction of managed competition was turned down in a national referendum and eventually abandoned, the CCS was no longer pursued either and discontinued by 2008. Although the existing 16 CCS care coordinators (6 hospitals, 5 polyclinics, and 5 GP groups) supported the continuation of the model, both the government and the opposition were against the CCS which allowed care coordinators to retain achieved savings that were coming from an already tight health care budget.

Similar ideas were later pursued under the term “health coordination” (to make it distinct from the original “care coordination” and thus politically acceptable) within a European Union (EU) funded project implemented in 2014–2015. A conceptual framework for reintroducing care coordination was developed, complemented with health coordination guidelines and protocols. Health coordination offices were to be established at the level of micro regions, each covering approximately 50,000 inhabitants. These offices were meant to be initially separate, but the plan was to eventually locate them within health care providers (like in the CCS model) once the implementation of the model was approved. Unfortunately, another change in government meant that the proposals were again shelved. Some of these ideas were picked up in the primary care reform, which started in 2013 (see below), but these were much less far reaching compared to the “care coordination” and “health coordination” models described above.

## Efforts to strengthen primary care

In all four countries, reforms aimed at strengthening PHC have been high on the policy agenda since the fall of Communism (9). Internationals agencies, such as the World Bank, the World Health Organization (WHO), and to a lesser extent the European Commission (EC) and bilateral donors, supported several initiatives designed to strengthen PHC in the region. Reforms of PHC continue, recently focusing more – at least in some countries – on shifting away from the solo practices and toward larger, multidisciplinary practices, which to some extent resembles the polyclinics model from the Soviet era (18).

The cornerstone of the primary care reform in Hungary, which was piloted between 2013–2017 with funding from the Swiss Contribution, was the horizontal integration of solo GP practices (usually composed of a doctor and a nurse) into group practices, comprising six or more single practices and with the additional involvement of other health professionals, such as dieticians, physiotherapists, public health experts and health mediators (19, 20). The integrated group practices retained their financial and organizational independence, but received extra funding to employ additional health professionals and provide more preventive services. The initiative was continued after the pilot ended in 2017, and in 2021 a national rollout started, also supported with additional funding. Pediatricians and dentists are now also allowed to join the group practices and higher funding is available for practices that opt for closer cooperation. The latter involves preparing a competency map and developing a plan for improving skills, equipment, and infrastructure in the practice, and providing additional surgery hours dedicated to prevention, and extra services, such as diagnostic tests, screening, and telemedicine. Case management and complex disease management programmes are not yet part of this initiative.

In Slovakia, a complex primary care reform plan was introduced in 2014 with the goal of overhauling the organization of primary care by 2030 (8). The plan was based on a proposal by the EC's Expert Panel that recommended to establish larger PHC teams or networks, resembling smaller polyclinics. These were referred to as Integrated Care Centers (ICCs) and were meant to integrate providers of outpatient care services by physically bringing them under one roof. This included PHC physicians, dentists, and gynecologists, at the minimum, and could also include other specialists and providers of social and public health services, depending on local needs. The Centers thus have the potential to become a one-stop shop for primary care services including basic diagnostic, preventive and social care services, providing continuous care for chronic patients and easing the burden on acute care hospitals (8). So far, progress has been slow, not least because practical implementation details have not been worked out. Eventually, in 2022, the Ministry of Heath developed a concept document for primary care, outlining the roles, processes (including coordination of tasks), competencies, and education of PHC doctors and nurses, financing and payment mechanisms, to provide the basis for strengthening of PHC in Slovakia. Once this document has been formally approved, implementation details will be progressively specified in dedicated guidelines.

An ambitious model of PHC reorganization was piloted in Poland between 2018 and 2021. The pilot, partly funded by the World Bank, sought to support development of multidisciplinary PHC teams, that besides a doctor and a nurse were to also include health educators, dieticians, and physiotherapists. While integration of solo GP practices into group practices (like in Hungary and Slovakia) was not explicitly encouraged, the pilot was more suited to larger practices, e.g., in terms of having established collaborations with specialist and in terms of ICT infrastructure, and few smaller practices, which dominate the Polish PHC landscape, met the formal requirements to join the pilot. The new model put much emphasis on health promotion and disease prevention, not only by including health educators and dieticians in PHC teams, but also by introducing periodic check-ups for qualifying registered patients (21). It also sought to increase the role of GPs in the management of chronic conditions by introducing DMPs for 11 most prevalent conditions in five areas (cardiology, diabetology, pulmonology, endocrinology, and rheumatology and neurology), including for diseases such as type 2 diabetes, chronic coronary heart disease, asthma, and COPD. It aimed to reduce referrals to specialists by allowing GPs to order extensive diagnostic and laboratory tests. If needed, GPs could consult with a range of cooperating specialists, while retaining the responsibility over the patient. Consenting patients would follow Individual Medical Care Plans that are tailored to their health condition(s) and are established jointly by the PHC team and the patient – the programme thus included elements of case management too (the top the Kaiser Pyramid; see Box 1). PHC teams were made responsible for coordinating patients' care pathways, including post-hospital treatment, with a new role of care coordinator introduced to that end. After the pilot was concluded, a national rollout was not pursued but the tested solutions are instead being implemented gradually and on a partly voluntary basis. Thus, in December 2021, all PHC practices were mandated to hire care coordinators, and in July 2022 the competencies of PHC doctors were extended to allow them to order a larger range of diagnostic tests. In October 2022, voluntary care coordination was introduced in four areas (as above but without rheumatology and neurology), with improved access to diagnostics and specialist consultations in these areas. This is in line with the World Bank's recommendations and is hoped to allow less-ready practices to learn from early implementers.

Poland also has various complex care programmes aimed at improving coordination of care for various diseases or groups of patients, which have been implemented over the past 15 years [see Table 1 in (22)]. However, some of these programmes are quite narrow, focusing mainly on diagnostics and specialist care, with only a few encompassing prevention and primary care services, or social care. The National Oncology Network, piloted since 2019, and the National Cardiology Network, piloted since 2021, have the ambition to offer a comprehensive range of services, from primary prevention to care for the most complex patients in their respective clinical areas, and to concentrate provision of highly specialist services in order to improve their quality. However, the role of PHC in the Oncology Network pilot has so far been minimal, even though many cancer patients in Poland are diagnosed too late to be successfully treated (23).

In the 2000's, an initiative that was similar to the Hungarian CCS model emerged in Czechia, but on a much lower scale than in Hungary, and showing more resemblance to UK's GP fundholding. Some regionally based health insurers gave their contracted GPs financial responsibility for selected, mainly outpatient health services consumed by their patients. The GPs were given virtual budgets to manage and could retain part of the savings if their average cost per patient was lower than the insurer's risk-adjusted average cost per member. These so-called “managed care projects” were administered by private parties contracted by the health insurers, and as part of the managed care support, GPs received regular feedback on their patients' care consumption and their own prescribing behavior, including how they compared to other GPs. The project had no central support and since it was not easy to achieve savings (the risk-adjustment formula on which the GP budgets were based was not well developed, and since there is not gatekeeping in the Czech system, patients could opt to see a specialist other than the one they were referred to by their GP) most participating health insurance funds have abandoned it by late 2000s. Nevertheless, some insurers still use benchmarking to compare their contracted GPs.

More recently, the Czech Ministry of Health has prioritized promoting primary care services by broadening the competencies of the GPs. Thus the remit of primary care has been progressively expanded, and since 2019 Czech GPs have been made responsible for patients with stabilized type 2 diabetes and patients who have recovered from cancer, and since 2020 for pre-diabetes care and early dementia detection. Provision of prevention, including vaccinations, and screening are also being incentivised with fee for service (FFS) payments, which now account for close to 40% of GPs' incomes (24), while the capitation rate has been progressively increased since the late 1990s.

Similarly to Poland, Czechia has also sought to concentrate provision of highly specialized care. Between 2008 and 2011, dedicated care networks for cancer, stroke, and cardiovascular disease patients were established, mainly covering specialist inpatient care (24, 25), and these have been further improved over the past decade. For example, Regional Oncology Groups were set up in 2017 to improve provision of cancer care, and the previously established Comprehensive Oncology Centers were charged with coordinating the full spectrum of cancer care within their regional group, including palliative care and home care services. Since 2019 the GPs have been included in these regional networks, after gaining responsibility for recovered cancer patients (see above) (24).

## Care coordination as a strategic objective

Improving coordination of care is a relatively new objective in the strategic health system documents in the four countries. In Poland, it was only recognized as a strategic goal in 2021. The strategic framework document titled “Healthy Future. A Strategic Framework for the Development of the Health Care System for 2021–2027, with a perspective until 2030” (26) postulates establishing new models of coordinated care, including for older people and for people with mental health conditions, and structures, such as the National Oncology Network and the National Cardiology Network. Introduction of care coordinators within PHC, was also explicitly mentioned and it was already implemented at the end of 2021.

In Czechia, improved care coordination is one of the strategic goals of the Strategic Framework for the Health Care Development titled “Health 2030”, which was first adopted by the Czech government in 2019 and later updated in 2020 in response to the COVID-19 pandemic (27). The Framework has seven priority areas, including continuation of the ongoing PHC reforms described above, implementation of coordinated care models, integration of health and social care, and development of community mental care.

In Slovakia and Hungary, care coordination was prioritized some years earlier – in 2013 in Slovakia and in 2011 in Hungary. Like now in Czechia, the main priority in Slovakia was to strengthen the role of PHC (28). The current government manifesto, approved in 2020, also supports improving coordination of care, including between health and social care sectors (29). In Hungary, the Semmelweis Plan from 2011 (30) was the first comprehensive strategic health policy document that addressed the question of the management of patient pathways across service providers. Interestingly, the earlier CCS reform has been a “stealth” reform, initiated by a wealthy businessman from a small town near Budapest, and later taken up by the Ministry of Health (31). However, with the exception of the PHC reform piloted in 2013–2017, which is currently being rolled out at the national scale, efforts to improve care coordination have been largely abandoned after 2014.

## Discussion and conclusions

The concept of health systems resilience and how to improve it in practice have recently received increased attention both among health systems analysts and policy makers. This is mainly due to the occurrence of sudden and acute system shocks, such as the financial crisis and the COVID-19 pandemic, but more gradual strains and stresses, such as the rise of multiple chronic conditions, will also affect health systems resilience over time. Nevertheless, adapting to the changing disease patterns is by no means straightforward for several reasons. First, the fast pace of technological development demands increased specialization, while patients with multiple chronic conditions require integrated service provision across types and levels of care and sectors, such as health and social care. Second, care coordination is an elusive concept, with multiplicity of (often overlapping) terms, and its analysis is often overcomplicated with very broad frameworks, such as the one developed by the SELFIE (Sustainable integrated chronic care modeLs for multi-morbidity: delivery, FInancing and performancE) project (https://www.selfie2020.eu). Much simpler frameworks, such as the Kaiser Triangle used in this Perspective, can help map the various reforms by focusing on which population groups and health needs these initiatives attempt to cover. Third, long-term changes such as care coordination require long-term planning, and this is often obstructed by election cycles and politics. Fourth, fiscal decision makers often focus on achieving short-term cost savings; however, improving care coordination, while potentially decreasing costs by eliminating unnecessary services and reducing avoidable hospitalisations, can also increase health care costs by uncovering and addressing previously unmet needs. Focusing more on primary prevention, as the Kaiser model and some of the more comprehensive care coordination programmes do, can decrease the costs, albeit only in the (very) long term. Fifth, health care is a very complex and politically sensitive area from the perspective of high-level decision makers, and there seems to be a reluctance among the CEE politicians to experiment with new ideas, which have not been tried in other, more affluent countries in the West.

These are only some of the factors behind the mixed picture that we have found while analyzing coordinated care initiatives in Czechia, Hungary, Poland, and Slovakia. The former communist countries of Central and Eastern Europe are often considered to be lagging behind Western Europe in terms of health reforms, but the efforts we have identified here show that it is not the lack of innovative ideas that set health systems in the East and West apart, but rather the political and technical feasibility of the pursued policies. While Hungary and Poland have pursued more far-reaching coordinated care initiatives compared to Czechia and Slovakia, these efforts have so far failed. This was because of politics and changed priorities, or because the reform was too ambitious for the realities on the ground, such as the PHC PLUS reform in Poland, which was unsuited for small PHC practices that dominate in the country. Perhaps consulting with the Polish PHC doctors on the details of the reform would have helped achieve a more realistic pilot. In Czechia for example, the successful implementation of increased GP competencies has been ascribed to the efforts by the Ministry of Health to secure support from the professional association and medical societies affected by the reform.

Financial reasons have likely also played a role: health spending in all four countries is comparatively low among the EU countries (both in per capita terms and as %-age of GDP), and relatively little is spent on PHC, with the latter partly a reflection of the dominance of specialist care but also of the low numbers of PHC physicians. However, positive developments have been noted, both in terms of increasing the overall health spending and the remuneration and financial incentives for primary care doctors.

All four countries continue to pursue incremental reforms to strengthen PHC, and although these may be less far reaching, they nevertheless have potential to make a big difference to the lives of patients suffering from chronic diseases and prepare the ground for more ambitious reforms. Efforts to concentrate provision of specialist care in dedicated networks pursed in Czechia and Poland also have the potential to involve PHC providers and further bolster PHC in these countries. Strengthening care coordination has been recognized as a strategic priority in all four countries and the Recovery and Resilience Facility that was set up to aid EU Member States in the recovery from the COVID-19 pandemic, can now be used to support the realization of this goal. It is now a good time for Czechia, Hungary, Poland, and Slovakia to take stock of what has been tried so far, including revisiting the failed initiatives and reforms pursued in other countries, and use it as an opportunity to better prepare to meet the changing health needs of their populations. Paying more attention to the technical and political feasibility of innovative ideas could be the game changer for future health reforms.

## Data availability statement

The original contributions presented in the study are included in the article/supplementary material, further inquiries can be directed to the corresponding author.

## Author contributions

Conceptualization: AS. Resources and writing–original draft preparation: AS, IK-B, LB, MS, IC, and PG. Revisions: AS, LB, MS, and PG. Supervision: PG. All authors contributed to the article and approved the submitted version.
